# Peripherally Inserted Central Catheter (PICC) placement in newborns, Italian training program for nurses: preliminary results

**DOI:** 10.1186/1824-7288-41-S1-A21

**Published:** 2015-09-24

**Authors:** Memoli Maria Rosaria, Aversa Salvatore, Motta Mario, Romitti Maria Grazia, Certi Marina, Previdera Roberta, Titone Carmela, Codega Alessandra, Marzollo Roberto, Pezzotti Elena, Dioni Elisabetta, Chirico Gaetano

**Affiliations:** 1 Neonatal Intensive Care Unit Children Hospital, Civil Hospital of Brescia, Italy

## 

In the Italian Neonatal intensive care Units the positioning of Peripherally Inserted Central Catheters (PICC) is strictly a medical competence, neonatal nurses are the ones fully entrusted of the PICCsmanagement. In 2014 a group of neonatologists and nurses established at the Civil Hospital in Brescia a PICC group; the aim was to review and update the internal procedures but, also to ponder the idea of training nurses to place PICCs. Many were the reasons for this last consideration; to improve the quality and safety of neonatal critical care, to standardize the procedures to reduce errors and complications but also to make neonatal nurses fully aware of their role in PICCs correct management. A course was organized in two phases (lessons and practice) with the aim to provide participants with the knowledge and skills to safely position and manage PICCs. A careful work of review of national and international scientific literature regarding PICCs was carried out, the NICU procedures were updated prior the course onset [1-5]. All the specific topics regarding PICCs were tackled during the first part of the course(theoretical). The second part (practical) was divided in three parts:A) “executor” nurses were supposed to successfully position 4 PICCs under medical supervision, B) “tutor” nurses were asked to correctly position 10 PICCs, C) maintenance of the acquired skills. Data collection forms have been designed to keep records on PICCs placements, management and potential adverse events. Of the 54 nurses working in NICU, 32 (59%) participated actively at the project. At present 20 nurses have started the practical training:6 have completed the A) phase.53 PICCs have been successfully placed by nurses. The medium number of PICC placement for nurse has been 2,65. The training course for nurses has shown a good safety profile, a high success rate, and no post-procedural complications; a questionnaire has been proposed to all the NICU staff to acknowledge their opinions and impact. These preliminary data are promising, but we need to complete the whole program before being able to encourage other centers to follow our steps.

## References

[B1] Pittiruti M Central venous catheters in neonates: old territory, new frontiers. Invited commentary to peripherally inserted central venous catheters in critically ill premature neonates, by Ozkiraz et al J Vasc Access 2013 14 4 320 324 J Vasc Access. 2013;14(4):318-319 10.5301/jva.5000157 23817952

[B2] Pain Study Group of the Italian Society of Neonatology Lago P Guidelines for procedural pain in the newborn ActaPaediatr 2009 98 6 932 939 10.1111/j.1651-2227.2009.01291.x PMC2688676 19484828

[B3] Ramasethu J Complications of vascular catheters in the neonatal intensive care unit ClinPerinatol 2008 35 1 199 222 10.1016/j.clp.2007.11.007 18280883

[B4] Sellitto M Messina F Central venous catheterization and thrombosis in newborns: update on diagnosis and management J Matern Fetal Neonatal Med 2012 25 Suppl 4 26 28 22958007 10.3109/14767058.2012.714974

[B5] Moureau N Lamperti M Kelly LJ Dawson R Elbarbary M van Boxtel AJ Pittiruti M Evidence-based consensus on the insertion of central venous access devices: definition of minimal requirements for training Br J Anaesth 2013 110 3 347 356 10.1093/bja/aes499 23361124

